# DRBD3 regulates long non-coding RNA abundance and cryptic splice site selection in trypanosomes

**DOI:** 10.1007/s00018-025-05929-w

**Published:** 2025-11-06

**Authors:** Gloria Ceballos-Pérez, Claudia Gómez-Liñán, Francisco J. Sánchez-Luque, José M. Pérez-Victoria, Antonio M. Estévez

**Affiliations:** Instituto de Parasitología y Biomedicina ‘‘López-Neyra’’, IPBLN-CSIC. Parque Tecnológico de Ciencias de la Salud, Avda. del Conocimiento, 17. 18016, Armilla, Granada Spain

**Keywords:** Trypanosoma brucei, LncRNAs, *trans*-splicing, *cis*-splicing, RNA-binding proteins, Cryptic splice site

## Abstract

Trypanosomes are unicellular eukaryotes that rely heavily on post-transcriptional mechanisms to control gene expression. DRBD3 is an RNA-binding protein known to play important roles in mRNA processing, stability, transport and translation. It was found to associate with *grumpy*, a long non-coding RNA (lncRNA) recently characterized in *Trypanosoma brucei*. Here, we explore the role of DRBD3 in lncRNA metabolism and show that its depletion leads to the upregulation of a specific subset of approximately one hundred lncRNAs in both bloodstream and procyclic forms, likely through the activation of cryptic splice sites. The effect of DRBD3 depletion on lncRNA expression appears to be mostly indirect, and results from reduced levels of the poly(A) polymerase PAP1 following DRBD3 silencing. In addition to its impact on lncRNAs, DRBD3 loss also affects the processing of protein-coding genes, leading to alternative *trans*-splicing and protein truncation. Furthermore, we demonstrate that DRBD3 regulates the splicing of the newly identified intron in the transcript encoding the RNA-binding protein RBP20, and is important for maintaining the balance between *trans*- and *cis*-splicing. Our results position DRBD3 as a high-level regulatory factor that shapes the expression landscape of both coding and non-coding genes in trypanosomes.

**Supplementary Information:**

The online version contains supplementary material available at 10.1007/s00018-025-05929-w.

## Introduction

Trypanosomatids comprise numerous parasitic trypanosomes and *Leishmania* species, which are responsible for serious diseases that threaten human health and hinder development worldwide [1]. These early-branching unicellular eukaryotes are considered powerful model organisms for investigating post-transcriptional regulation, since they rely heavily on mRNA processing, stability and translation to control gene expression [2]. This is due to the unconventional organization of their genomes in long polycistronic transcription units that are transcribed constitutively, leaving little room to conventional transcriptional regulation [3]. Individual, mature mRNAs are generated by *trans*-splicing of a capped spliced leader sequence at the 5’-end, and coupled polyadenylation at the 3’ end [4]. Notably, only three transcripts undergo *cis*-splicing in trypanosomes [5, 6], all encoding nuclear proteins with known or predicted roles in RNA metabolism: poly(A)-polymerase 1 (PAP1, Tb927.3.3160), the RNA-helicase DBP2B (Tb927.8.1510) and the RNA-binding protein RBP20 (Tb927.8.6440). Although cross-talk between *cis*- and *trans*-splicing has been proposed [4, 6], the mechanisms underlying such interplay remain poorly understood.

*Trypanosoma brucei* is responsible for human and animal trypanosomiasis in sub-Saharan Africa [7]. Trypanosomes are transmitted between mammals by tsetse flies, and undergo a profound differentiation process in order to adapt to the different environments they face in the life cycle [8]. Two life stages are easily cultivated in the laboratory: procyclic (insect) forms and bloodstream (mammalian) forms. In addition to major morphological and metabolical changes, bloodstream trypanosomes replace the variant surface glycoprotein (VSG) with two insect-specific coat proteins known as GPEET and EP procyclins, who play important roles in parasite survival [9].

As anticipated in organisms with limited transcriptional regulation, RNA-binding proteins play a crucial role in determining the final abundance of mRNAs and proteins [3]. Double RNA-binding domain 3 (DRBD3) is a nucleocytoplasmic RNA-binding protein that plays a pivotal role in post-transcriptional gene regulation in *T. brucei* [10, 11]. DRBD3 has two RNA recognition motifs (RRMs), and it is highly conserved among trypanosomatids ([10, 12, 13], https://tritrypdb.org). It binds to specific subsets of mRNAs, including those encoding ribosomal proteins, translation factors, membrane proteins, and enzymes involved in proline catabolism [10, 11, 14]. DRBD3 stabilizes its targets and modulates their translation, thereby influencing energy metabolism and protein synthesis. Under environmental stress, DRBD3 relocalizes to stress granules or the nucleus and forms dynamic ribonucleoprotein complexes [15]. In addition, DRBD3 has been proposed to regulate both *cis*- and *trans*-splicing [11]. Notably, it inhibits *trans*-splicing when tethered upstream a reporter gene [16], an observation that, together with protein homology analyses, suggests that DRBD3 is the trypanosome homologue of mammalian polypyrimidine-tract-binding protein 1 (PTB1 [11, 16]). All these features position DRBD3 as a key coordinator of gene expression in trypanosomes.

Long non-coding RNAs (lncRNAs) are a diverse class of transcripts longer than 200 nucleotides that do not encode proteins but play important roles in regulating gene expression [17]. They function through various mechanisms, including modulation of chromatin structure, transcription, RNA processing, and translation. LncRNAs can act in *cis* or *trans*, and often associate with RNA-binding proteins or other RNAs to exert their effects [18]. Although best characterized in mammals, lncRNAs have been identified across eukaryotes, where they contribute to development, stress responses, and disease [19]. Their functions in early-diverging organisms like trypanosomes, however, remain largely unexplored. Until recently, the repertoire of identified lncRNAs in trypanosomes was limited, and virtually nothing was known about their roles in parasite biology. However, recent transcriptomic surveys have uncovered a large number of lncRNAs in both trypanosomes and leishmanias [20–24], which are also processed by *trans*-splicing and polyadenylation, and possess poly(A) tails that are shorter than those of mRNAs [20]. In *T. brucei*, two lncRNAs, named *grumpy* and *TblncRNA-23*, have been characterized in some detail, with demonstrated roles in cell differentiation and social motility [20, 25]. Pulldown assays using *grumpy* as bait identified DRBD3 as a specific interactor, a finding confirmed by DRBD3 immunoprecipitation experiments [20]. Given DRBD3’s established roles in post-transcriptional regulation in *T. brucei*, we investigated whether it also influences lncRNA expression. Our results show that DRBD3 regulates a defined subset of lncRNAs in both bloodstream and procyclic forms, primarily through modulation of cryptic splice acceptor site selection, revealing important roles for DRBD3 in both *trans*- and *cis*-splicing.

## Materials and methods

### Trypanosome culture and RNA interference

‘Single marker’ *T. brucei* Lister 427 bloodstream cell line S16 [26] was maintained in HMI-9 medium [27] containing 10% fetal bovine serum at 37 °C with 5% CO_2_. *Trypanosoma brucei* 449 procyclic cells [28] were cultured at 28 °C in SDM-79 medium [29] supplemented with 10% fetal bovine serum. Trypanosomes were transfected following standard procedures [30]. DRBD3 expression was silenced by RNAi using bloodstream and procyclic cell lines transfected with plasmid pGR69, which expresses dsRNA corresponding to the first 500 nt of the DRBD3 gene in a tetracycline inducible fashion [10]. Cells were incubated for 48 h in the presence of 1 µg/ml of tetracycline, and successful depletion of DRBD3 was confirmed by western blot assays in all RNAi experiments [10]. For RNAi of zinc finger protein 41 (ZC3H41) in bloodstream forms, plasmid pGR309 [31] was transfected in S16 trypanosomes and induced as above.

### RNA-seq

Total RNA was obtained from either untransfected (parental S16 or 449 cells) or from RNAi-induced cells (48 hours in the presence of tetracycline) using the RNeasy Mini Kit (Qiagen). Poly(A)-selected RNA libraries were prepared using the standard TruSeq Stranded mRNA sample preparation protocol (Illumina). Biological triplicates were sequenced at the Genomics Unit of the IPBLN-CSIC (Granada, Spain) using a NextSeq 500 platform (Illumina). The resulting 76-nt paired end sequences were checked for quality and mapped to the eleven megabase chromosomes of the *T. brucei* TREU927 (v6.8) genome as described [32]. Reads assignment to genes was done using the ‘featureCounts’ program of the Subread package (version 1.5.0-p [33]) with options -p -O -B -C; the annotation file was TriTrypDB-68_TbruceiTREU927.gff (downloaded from https://tritrypdb.org), modified to include 3’-UTRs annotations informed by nanopore sequencing [34] and lncRNAs coordinates [20]. Principal component analysis, splice-acceptor sites mapping and coverage profiles were done as previously described [32]. Split reads coordinates were extracted with the ‘subjunc’ program of the Subread package [35] and processed using the ‘intersect’ module of the bedtools package [36] with options -s -F 1.

External RNA-seq data were downloaded from the European Nucleotide Archive: bloodstream double RNA-binding domain 18 (DRBD18) RNAi, project PRJEB41419 [37]; procyclic DRBD18 RNAi (whole cells), PRJNA665716 [38]; ZC3H41 procyclic RNAi, PRJNA878472 [31]; procyclic PAP1 RNAi (5′ directed SL and poly (A)-selected libraries), PRJNA385386 [39]) and subjected to the same pipeline described above.

### PCR analysis

Quantitative RT-PCR reactions were carried out in 96-well pates (Thermo Scientific) using a BioRad CFX96 thermal cycler as described [15, 40]. Fold-changes in expression were calculated using the 2^−∆∆CT^ method [41] with *actin* mRNA as the reference. RNA immunoprecipitation followed by quantitative RT-PCR was performed using an anti-DRBD3 antiserum [10], with normal rabbit serum as a control and *actin* mRNA as the reference [15]. All quantitative RT-PCR experiments were conducted with three biological replicates. For assays of *RBP20* splicing, 4 µg of total RNA purified from procyclic trypanosomes were reverse transcribed using Maxima reverse transcriptase (Thermo Scientific) and the antisense primer AE1415, which hybridizes to the second exon. The resulting cDNA was purified with the Macherey-Nagel PCR purification kit and 1/30th was subjected to 25 cycles of conventional PCR using a sense primer AE1416 (hybridizing to the first exon), and antisense AE1417 (hybridizing to the second exon upstream AE1415). For 3’-nested PCR of polyadenylated PAP1 exon 1, total RNA was reverse transcribed as above using the anchored oligo(dT)_18_ primer CZ1584, which includes a 5’ tag [42]. The resulting cDNA was amplified in two rounds of PCR using nested sense primers AE1391 and AE1393 (hybridizing within exon 1) together with the antisense primer CZ1585, which hybridizes to the CZ1584 5’ tag. All oligodeoxynucleotides used in this study are listed in Table S1.

### Protein analysis and luciferase assays

Cell lines expressing 4xTy- or TAP-tagged proteins from their endogenous loci were generated using plasmids described in [43], and subsequently transfected with the RNAi plasmid pGR69 [10] for DRBD3 depletion. Tagged proteins were detected by western blot using either the BB2 monoclonal antibody [44] or anti-protein A antiserum (Sigma), and visualized by chemiluminescence or with an Odyssey CLx Near-Infrared Fluorescence Imaging System. Specific antisera against EP procyclins (Cedarlane), GPEET procyclin [45] and RNA-binding protein RBP10 [46] were also used. Quantifications were done using ImageJ [47] or Image Studio (version 5.2; LI-COR Biosciences). For luciferase reporter assays, S16 or 449 cell lines were transfected with plasmids based on pGR435, a pGR108-derivative [48] lacking T7 terminator sequences and bearing a luciferase gene flanked by the 3’UTRs under study. The resulting cell lines were further transfected with pGR69 as described above. Luciferase assays were carried out as in [48].

## Results

### Depletion of DRBD3 results in the altered expression of a specific subset of LncRNAs

To gain insight into the function of DRBD3 in lncRNA metabolism, we analyzed the transcriptomes of bloodstream and procyclic trypanosomes by high-throughput sequencing upon depletion of DRBD3 by RNAi. RNA-seq replicates showed good reproducibility, with pairwise Pearson correlation coefficients >0.990 and clear clustering in principal component analysis (Fig. S1A). Differential expression analysis confirmed the regulation of known DRBD3 targets [10, 11, 14], such as mRNAs encoding membrane transporters or proteins involved in translation (Fig. S1B and Tables S2 and S3).

**Fig. 1:**
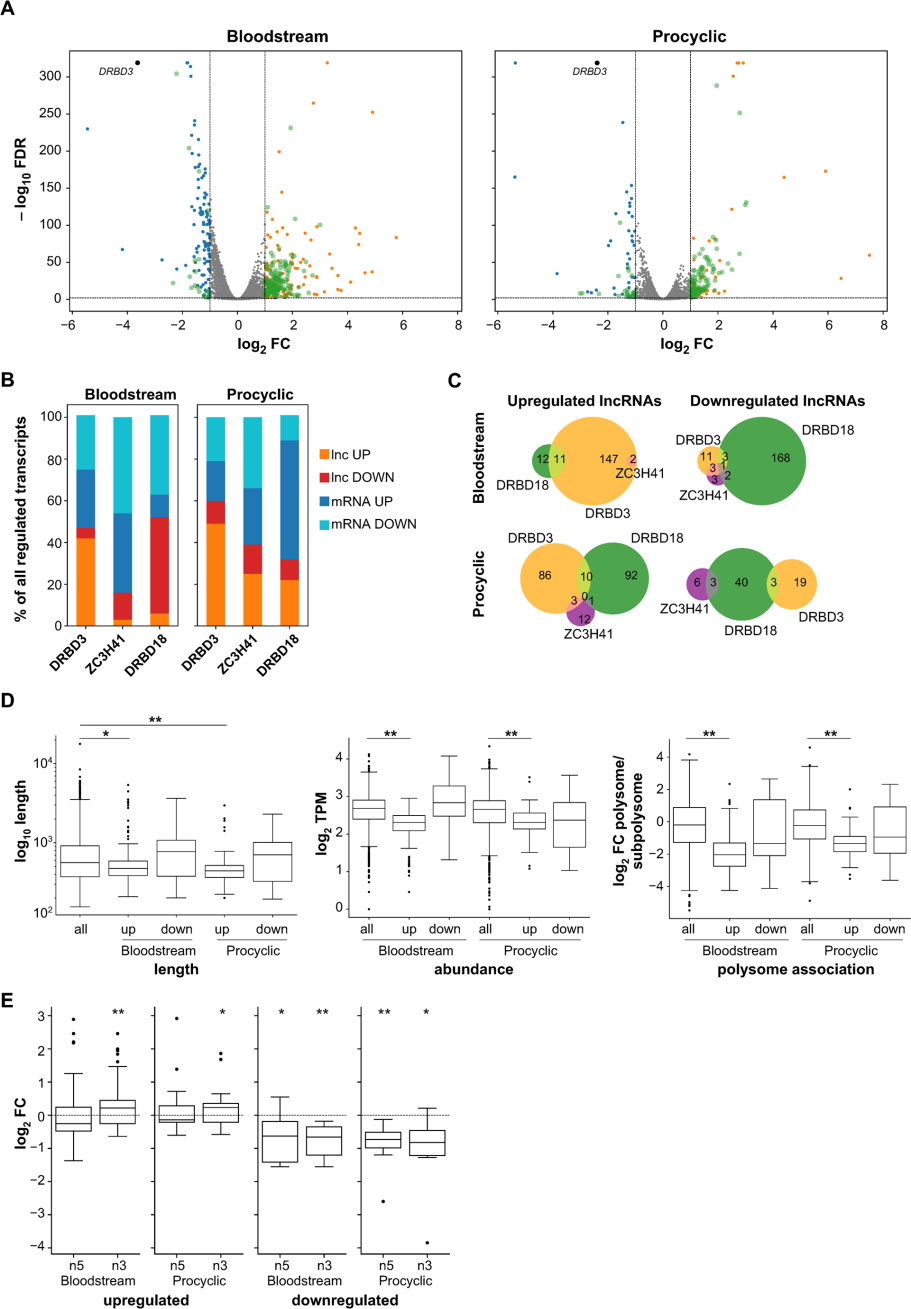
Effects of DRBD3 depletion on the abundance of lncRNAs. **A** Volcano plot of differential gene expression in DRBD3-depleted bloodstream and procyclic trypanosomes compared to control cells. Blue dots correspond to downregulated mRNAs, orange dots indicate upregulated mRNAs and green dots represent lncRNAs. DRBD3 transcript is shown as a black circle. Thresholds for differential abundance were |log_2_ fold change (FC)|≥ 1.0 and false discovery rate (FDR) ≤ 0.01. **B** Bar plot indicating relative proportions of up- and downregulated mRNAs and lncRNAs in DRBD3-, ZC3H41- and DRBD18-depleted cells. **C** Venn diagram of regulated lncRNAs detected upon depletion of DRBD3 (orange), ZC3H41 (purple) and DRBD18 (green). Numbers indicate unique and common lncRNAs between the datasets. **D** Boxplots representing length, abundance and association with polysomes of DRBD3-regulated lncRNAs (boxes, IQR; waists, medians; whiskers, ± 1.5 IQR; dots, outliers). TPM, transcripts per million; up, upregulated lncRNAs; down, downregulated lncRNAs. Two-tailed Mann–Whitney U-tests were used to assess whether there were significant differences between regulated and all annotated lncRNAs. *, *p* < 5 × 10^−3^; **, *p* < 5 × 10^−5^. No significant differences were observed for downregulated lncRNAs (all *p* >0.1). Polysome association data was taken from [20]. **E** Expression changes in genes adjacent to regulated lncRNAs. Boxplots represent log_2_ fold-change values (logFC) of 5´-neighbor (n5) and 3´-neighbor (n3) protein-coding genes. Two-tailed, one-sample t-tests assessed whether mean values differed significantly from log_2_ = 0 (no expression change, dashed line). *, *p* < 5 × 10^−2^; **, *p* < 5 × 10^−3^

A volcano plot for the differentially expressed genes in DRBD3-depleted trypanosomes is shown in Fig. 1 A, with lncRNAs highlighted in green. Between 8% (procyclic forms, 121 out of 1491) and 12% (bloodstream forms, 178 out of 1491) of all annotated lncRNAs varied significantly in abundance, with upregulated lncRNAs representing the predominant class in both life cycle forms (40–50% of all regulated transcripts, Fig. 1B).

To evaluate the specificity of DRBD3-dependent lncRNA regulation, we analyzed published transcriptomes of DRBD18-depleted trypanosomes (bloodstream and procyclic forms) and ZC3H41-depleted procyclic forms. DRBD18 is an essential nucleocytoplasmic RNA-binding protein involved in mRNA processing, transport and translation [37, 38, 49, 50], whereas ZC3H41 is a cytoplasmic RNA-binding protein required for vesicular RNA transport, translation, and rRNA processing [31, 51]. For bloodstream comparisons, we generated a ZC3H41 RNAi cell line and analyzed the transcriptome by RNA-seq (see Materials and Methods); ZC3H41 is essential in both life cycle forms ([31] and Supplementary Fig. 1 C). A total of 197 (bloodstream) or 149 (procyclic) lncRNAs showed differential expression in DRBD18-depleted trypanosomes, whereas only 11 (bloodstream) or 25 (procyclic) lncRNAs were found to be regulated in the ZC3H41 dataset (Fig. 1B and Tables S4 to S7). Compared to DRBD3, the proportion of upregulated lncRNAs relative to all differentially expressed transcripts was markedly lower in cells lacking DRBD18 or ZC3H41 (Fig. 1B). Moreover, there was little overlap between regulated lncRNAs in the three datasets (Fig. 1 C). These results indicate that the observed effects of DRBD3 silencing on the abundance of lncRNAs are specific.

There was a moderate correlation (Pearson’s *r* = 0.656) in lncRNAs fold-change values between bloodstream and procyclic DRBD3 datasets (Fig. S1D), suggesting that overall DRBD3-dependent modulation of lncRNA abundance is similar in both life cycle forms. Some lncRNAs, however, exhibited stage-specific control, showing significant abundance changes in one life cycle forms but not in the other, or even inverse regulation in at least six cases (Table S8). Notably, the only two lncRNAs functionally characterized so far in *T. brucei*, *grumpy* (*KS17gene_3137a* [20]) and *TblncRNA-23* [25] (named *KS17gene_1079a* in [20]) were both upregulated in DRBD3-depleted bloodstream and procyclic trypanosomes (Tables S2 and S3, and see below).

We next analyzed DRBD3-regulated lncRNAs in more detail, and observed that upregulated lncRNAs are significantly shorter, show reduced polysome association, and exhibit lower expression levels compared to all annotated lncRNAs, whereas downregulated lncRNAs showed no significant differences in these features (Fig. 1D). Given the established role of lncRNAs in modulating neighboring gene expression [19], we examined whether flanking protein-coding genes were similarly affected by DRBD3 depletion. Transcripts adjacent to upregulated lncRNAs were generally unaffected, whereas those flanking downregulated lncRNAs tended to decrease in abundance (Fig. 1E and Tables S9 and S10). This contrasted with DRBD18-depleted cells, where protein-coding genes flanking upregulated lncRNAs showed pronounced expression changes, particularly in procyclic forms (Fig. S1E), consistent with prior findings [50]. Protein-coding genes adjacent to DRBD3-regulated lncRNAs showed no significant enrichment in Gene Ontology terms (data not shown).

The coordinated regulation of protein-coding genes near downregulated lncRNAs may reflect their frequent overlap with untranslated regions (UTRs; Fig. S2). Indeed, 50–60% of downregulated lncRNAs are partially or fully embedded within mRNA UTRs (Supplementary Table 8), and RNA-seq coverage analysis confirmed that many of them represent segments belonging to longer mRNA transcripts (Fig. S2). Thus, their regulation seems to be linked to the expression of their overlapping mRNAs. In contrast, less than 10% of the upregulated lncRNAs overlap with annotated UTRs (Table S8). These overlap percentages (for both down- and upregulated lncRNAs) are probably underestimates, as 3’-UTRs frequently extend beyond annotated boundaries (see below).

## Regulation of LncRNA processing and neighboring gene expression

**Fig. 2:**
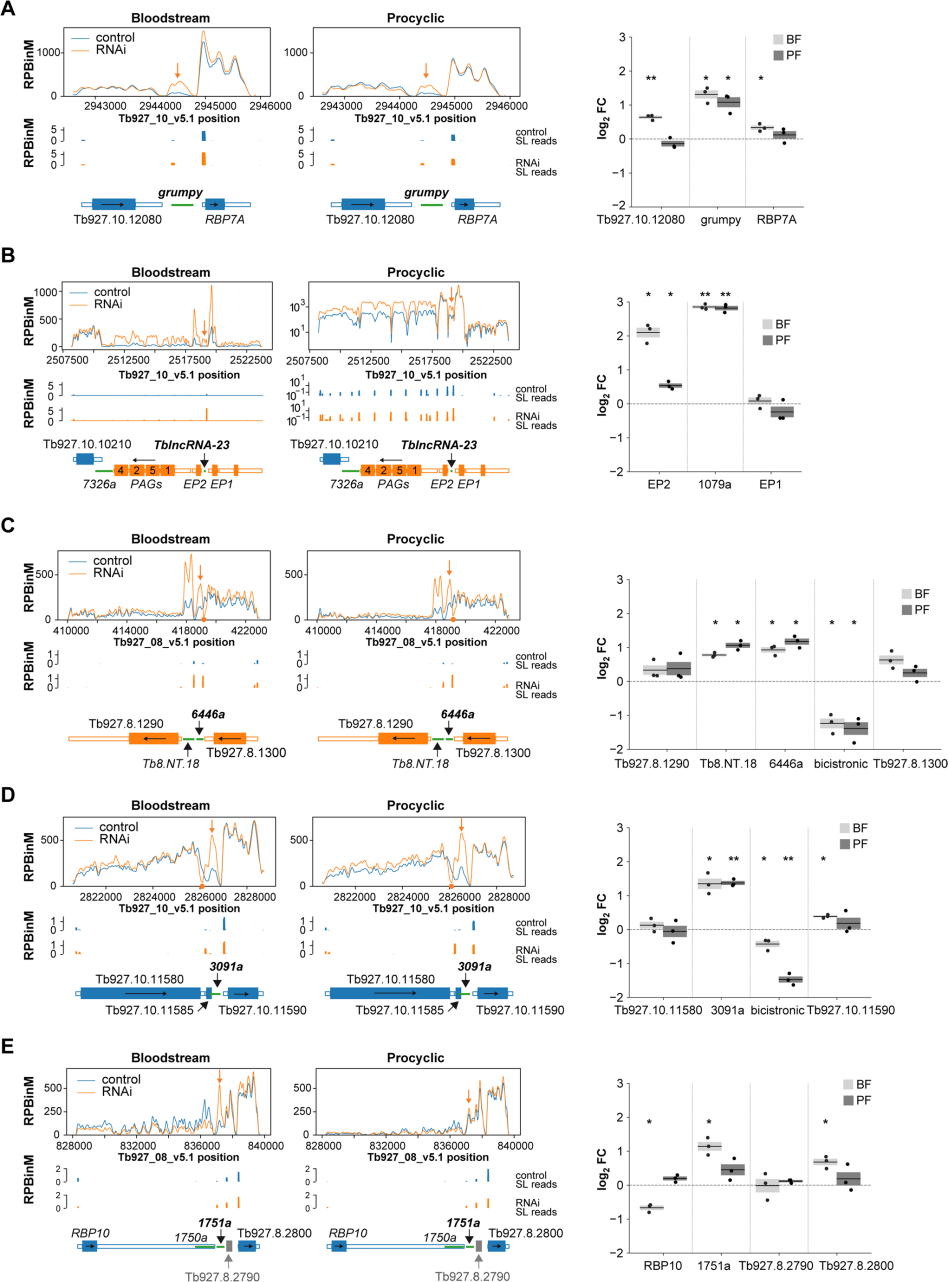
DRBD3-dependent regulation of representative lncRNAs. **A** *grumpy*; **B** *TblncRNA-23*; **C** *KS17gene_6446a*; **D** *KS17gene_3091a*; **E** *KS17gene_1751a*. Left panels, RNA-seq coverage plots of lncRNAs and genomic neighbors in control (blue) or DRBD3-depleted (orange profiles) bloodstream and procyclic trypanosomes. Average read counts across replicates were obtained using sliding windows (bin size, 100 bp; step, 10 bp) and normalized to library size (RPBinM, reads per bin per million mapped reads). Profiles corresponding to reads containing the spliced-leader sequence (SL) are also shown. Open-reading frames are represented as thick, filled boxes, while untranslated regions are depicted as thin, empty boxes (blue, genes in the Watson [+] strand; orange, genes in the Crick [–] strand). Long non-coding RNAs are shown as thin green boxes, and pseudogenes as thick grey filled boxes. Black arrows indicate the direction of transcription. Relevant lncRNA names are highlighted in bold, and their positions indicated by orange vertical arrows above the corresponding coverage profiles. RPBinM values for *TblncRNA-23* procyclic coverage plots are displayed on a log_10_ scale. Right panels, validation of lncRNA and neighboring gene expression changes upon DRBD3 depletion by quantitative RT-PCR. Fold changes (FC, log_2_ converted) relative to control of three independent RNAi inductions in bloodstream (BF) or procyclic (PF) cells are expressed as the mean (horizontal solid lines) ± s.e.m. (shaded areas). The effect of DRBD3 depletion on alternative *trans*-splicing (‘bicistronic’) of Tb927.8.1300-*KS17gene_6446a* (C) or Tb927.10.11585-KS17gene_3091a transcripts (D) was assayed using oligonucleotides flanking the alternative splicing-acceptor sites (locations marked by orange circles in coverage plots). Two-tailed, one-sample t-tests assessed whether mean values differed significantly from log_2_ = 0 (no expression change, dashed line). Only statistically significant changes are marked (*, *p* < 5 × 10^−2^; **, *p* < 5 × 10^−3^)

We focused subsequent analyses on five upregulated lncRNAs, chosen based on their roles in trypanosome biology, proximity to known regulators of gene expression, or the presence of distinctive read coverage profiles upon DRBD3 silencing: *grumpy*, *TblncRNA-23*, *KS17gene_6446a*, *KS17gene_3091a* and *KS17gene_1751a*. RNA-seq coverage profiles for these lncRNAs and their genomic neighborhoods are shown in Fig. 2. Profiles corresponding to reads containing at least 14 nucleotides of the spliced-leader sequence are also included to facilitate interpretation of transcript processing events, along with quantitative RT-PCR assays to assess changes in expression of lncRNAs and flanking transcripts in DRBD3-depleted cells.

*Grumpy* appears to be processed independently of its flanking protein-coding transcripts, Tb927.10.12080 and RNA-binding protein 7 A (*RBP7A*, Fig. 2 A). Upregulation of *grumpy* was confirmed by quantitative RT-PCR in DRBD3-depleted trypanosomes, whereas little or no changes were observed in the levels of adjacent transcripts. We observed a modest but significant upregulation of Tb927.10.12080 in bloodstream forms; this, however, did not result in increased levels of the corresponding protein product (see below). No changes were observed in the expression of *grumpy* locus in DRBD18- or ZC3H41-depleted cells, as judged by RNA-seq coverage profiles and the lack of significant changes in our differential expression analysis (Fig. S3 and Tables S4 to S7).

*TblncRNA-*23 is flanked by the procyclin genes *EP1* and *EP2* in chromosome 10, or procyclins *GPEET* and *EP3-2* in chromosome 6. No read coverage was detected in *TblncRNA-23* adjacent regions in bloodstream forms, whereas reads corresponding to the *EP1*-*TblncRNA-23* or GPEET-*TblncRNA-23* intergenic region were abundant in procyclic trypanosomes (Fig. 2B). Silencing of DRBD3 expression resulted in the overexpression of *EP2* and the four procyclin associated genes (PAGs) located downstream; PAG4 overexpression in DRBD3-depleted cells was observed in previous reports [10]. Small or no changes were observed in the expression of *TblncRNA-*23 or procyclins in DRBD18- or ZC3H41-depleted cells (Fig. S3).

The lncRNA *KS17gene_6446a* is flanked upstream by Tb927.8.1300 (encoding an uncharacterized flagellar pocket collar protein [52]), and downstream by another DRBD3-regulated lncRNA, *Tb8.NT.18*, and by Tb927.8.1290 (encoding a SUMO-interacting motif-containing protein, https://tritrypdb.org, Fig. 2 C). RNA-seq coverage analysis revealed abundant reads in the Tb927.8.1300-*KS17gene_6446a* and *Tb8.NT.18*-Tb927.8.1290 intergenic regions, with minimal coverage between *KS17gene_6446a* and *Tb8.NT.18* (Fig. 2 C). This is consistent with *KS17gene_6446a* being partially incorporated into the 3’-UTR of Tb927.8.1300 transcripts, while *Tb8.NT.18* may function as part of the 5’UTR of Tb927.8.1290. We could confirm the overexpression of *KS17gene_6446a* and *Tb8.NT.18* in bloodstream and procyclic DRBD3-silenced tyrpanosomes, while flanking transcript levels remained largely unchanged (Fig. 2 C). *Tb8.NT.18*, but not *KS17gene_6446a*, was also overexpressed in DRBD18-depleted procyclic cells, whereas neither lncRNA responded to ZC3H41 depletion (Fig. S4). Notably, DRBD3 silencing altered *KS17gene_6446a trans*-splicing patterns, as evidenced by distinct coverage profiles changes observed in DRBD3-depleted conditions (Fig. 2 C). This was confirmed by quantitative RT-PCR using oligonucleotides flanking the *KS17gene_6446a trans*-splicing acceptor site, which should amplify transcripts spanning both Tb927.8.1300 and *KS17gene_6446a* (Fig. 2 C). The significant depletion of these bicistronic transcripts in DRBD3-silenced trypanosomes suggests that DRBD3 normally prevents a *trans*-splicing event that would otherwise generate a truncated Tb927.8.1300 mRNA and accumulated levels of *KS17gene_6446a*. Alternative processing of *KS17gene_6446a* could also be observed in DRBD18-depleted cells, but to a lesser extent and in the absence of *KS17gene_6446a* accumulation, whereas no changes were evident in ZC3H41-silenced trypanosomes (Fig. S4).

A similar regulatory pattern emerged at the *KS17gene_3091a* locus. This lncRNA is flanked by Tb927.10.11580, encoding an uncharacterized WD40 repeat protein (https://tritrypdb.org), and Tb927.10.11585 (Fig. 2D). The latter gene probably corresponds to a lncRNA, since it encodes a small peptide that has not been detected in any proteomic survey, shows no significant similarity to known protein sequences in other kinetoplastid species, and partially overlaps with *KS17gene_3091a* gene (https://tritrypdb.org). RNA-seq coverage indicates that Tb927.10.11585 is partially embedded within the Tb927.10.11580 3’-UTR, while *KS17gene_3091a* transcripts are predominantly independently processed (although some transcripts encompassing all three species occur, Fig. 2D). Quantitative RT-PCR confirmed *KS17gene_3091a* upregulation in DRBD3-depleted cells, with minimal expression changes in flanking genes (Fig. 2D). DRBD3 silencing resulted in altered processing patterns, favoring Tb927.10.11585 *trans*-splicing at the expense of *KS17gene_3091a* excision (Fig. 2D). As in the case of *KS17gene_6446a* described above, a similar but less pronounced phenotype was observed in DRBD18-depleted cells, without evident concomitant *KS17gene_3091a* accumulation (Fig. S4).

We next characterized the regulation of *KS17gene_1751a* and its genomic neighbors RNA-binding protein 10 (*RBP10*), Tb927.8.2790 (a pseudogene encoding a truncated acetyl-CoA synthetase), and Tb927.8.2800 (encoding an uncharacterized flagellar protein). RNA-seq coverage patterns revealed that while *KS17gene_1751a* is primarily processed independently of *RBP10*, some transcripts spanning *KS17gene_1751a* and the downstream pseudogene Tb927.8.2790 were detectable; Tb927.8.2800 mRNA processing appeared unrelated to these upstream events (Figs. 2E). *KS17gene_1751a* was specifically upregulated in DRBD3-depleted bloodstream forms, accompanied by a moderate reduction in *RBP10* mRNA levels (Fig. 2E). Interestingly, the expression of *KS17gene_1750a*, another lncRNA embedded within the *RBP10* 3’-UTR, remained unchanged upon DRBD3 depletion but showed stage-specific regulation in DRBD18-deficient cells (decreasing in bloodstream while increasing in procyclic forms, Fig. S4). This pattern is in agreement with the *RBP10* mRNA processing defects observed upon DRBD18 depletion in bloodstream forms [37].

In summary, RNA-seq and qRT-PCR analysis showed that DRBD3 silencing consistently led to lncRNA upregulation, while adjacent mRNA levels were generally unaffected. For *KS17gene_6446a* and *KS17gene_3091a*, DRBD3 depletion specifically altered *trans*-splicing patterns, leading to the production of transcripts with truncated 3’-UTRs.

**Fig. 3:**
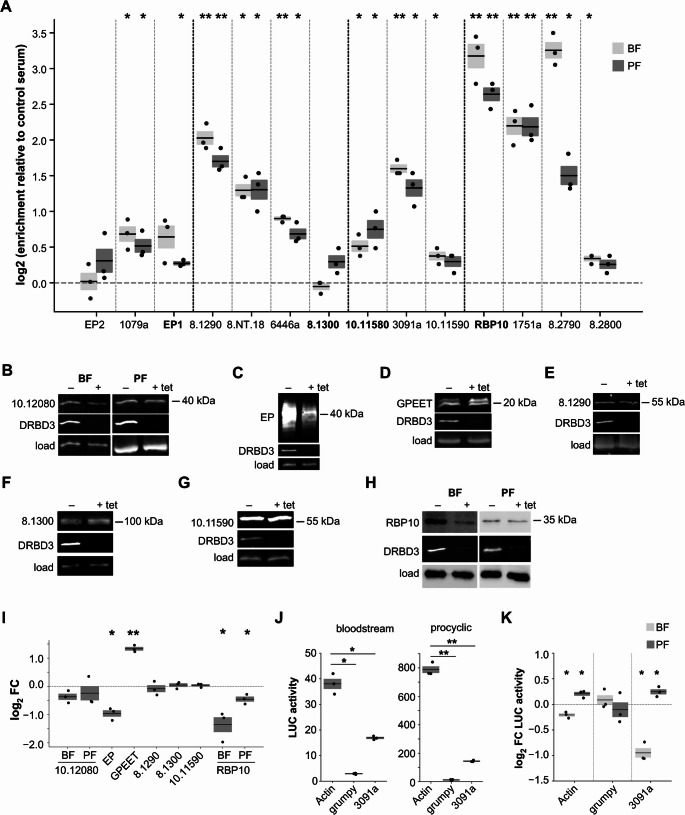
Association of DRBD3 with regulated lncRNAs and effect of DRBD3 depletion on the expression of neighboring genes. **A** RNA immunoprecipitation followed by quantitative RT-PCR. Fold-enrichment of target transcripts was calculated after comparing to mock immunoprecipitations carried out using normal rabbit serum and normalizing to *actin* mRNA. Values are expressed as the mean ± s.e.m. (*n* = 3). Transcripts located upstream of lncRNAs are highlighted in bold; transcript systematic identifiers are shown without the ‘Tb927.’ prefix. Two-tailed, one-sample t-tests assessed whether mean values differed significantly from log_2_ = 0 (no enrichment, dashed line). Only statistically significant changes are marked (*, *p* < 5 × 10^−2^; **, *p* < 5 × 10^−3^). **B-H** western blot analysis of 4xTy-tagged proteins encoded by neighboring genes in bloodstream (B and H) and/or procyclic extracts (B-H). α-tubulin (panels B -procyclic-, D and E), RRP4 ([53], panels B -bloodstream-, C, F and G) or p22 ([54], panel H) were used as loading controls. **I** quantification of 4xTy- tagged proteins levels in three independent experiments; *, *p* < 5 × 10^−2^; **, *p* < 5 × 10^−3^ (two-tailed, one-sample t-tests vs. log_2_ = 0, dashed line). **J** luciferase activity (expressed as millions of relative light units per mg of protein) was measured in bloodstream S16 or procyclic 449 cells expressing the reporter gene fused to an actin 3’-UTR (control), the Tb927.10.12080-RBP7A intergenic region (containing the lncRNA *grumpy*) or the Tb927.10.11580-Tb927.10.11590 intergenic region (containing Tb927.10.11595 and *KS17gene_3091a*). Values are expressed as the mean ± s.e.m. (n = 3), and were compared using two-tailed, independent t-tests (*, p < 5 × 10^−3^; **, p < 5 × 10^−5^). **K** effect of DRBD3 depletion on luciferase expression. Luciferase activity was monitored in RNAi-induced vs. uninduced cells in three independent experiments. *, p < 5 × 10^−2^ (two-tailed, one-sample t-tests vs. log_2_ = 0, dashed line)

To assess whether DRBD3 associates with these lncRNAs and their flanking transcripts, we performed native RNA immunoprecipitation (RIP) followed by qRT-PCR (Fig. 3 A). Previous work demonstrated DRBD3 binding to *grumpy* and its upstream neighbor Tb927.10.12080, but not the downstream RBP7A mRNA [10]. Our analysis showed that DRBD3 associates with all tested lncRNAs, albeit with varying RIP efficiencies. We could also confirm binding to upstream transcripts (highlighted in bold in Fig. 3 A), except in the case of Tb927.8.1300. We also detected DRBD3 binding to downstream transcripts (Tb927.8.1290 adjacent to *Tb8.NT.18* and the pseudogene Tb927.8.2790 flanking *KS17gene_1751a*). Combined with the RNA-seq coverage patterns shown above, these results suggest that *grumpy*, *TblncRNA-23*, *KS17gene_3091a* and *KS17gene_1751a* can be processed either as independent transcripts or as components of neighboring genes’ UTRs.

We showed above that the abundance of transcripts flanking regulated lncRNAs remained largely unaltered. To determine whether this was also the case at the protein level, we analyzed protein abundance upon DRBD3 depletion using Ty-epitope tagging [44] or available antisera. No significant changes were observed in Tb927.10.12080 (flanking *grumpy*, Fig. 3B and I), Tb927.8.1290 and Tb927.8.1300 (flanking *KS17gene_6446a*, Fig. 3E, F and I), or Tb927.10.11590 (flanking *KS17gene_3091a*, Fig. 3G and I). In contrast, procyclin EP (flanking *TblncRNA-23* in chromosome 10) decreased in abundance ~ 2-fold in DRBD3-silenced procyclic trypanosomes. Procyclin GPEET (flanking *TblncRNA-23* in chromosome 6) migrated as a doublet in SDS-PAGE gels, as previously described [45]; the slower-migrating band increased ~ 2.5-fold in abundance upon DRBD3 depletion (Figs. 3 C, D and I). EP and GPEET changes are reminiscent of, although more modest than, those observed during *TblncRNA-23* overexpression [25]. Lastly, depletion of DRBD3 resulted in a significant decrease in the abundance of RBP10 (flanking *KS17gene_1751a*), especially in bloodstream forms (Figs. 3 H and I).

The absence of DRBD3-dependent regulation of Tb927.10.12080 expression was confirmed using a luciferase reporter assay. Fusion of the Tb927.10.12080-*RBP7A* intergenic region (containing *grumpy*) to luciferase caused a dramatic inhibition of reporter activity (~ 13-fold in bloodstream and ~ 70-fold in procyclic forms) compared to a control cell line expressing luciferase fused to an actin 3’-UTRs (Fig. 3 J). However, DRBD3 depletion did not significantly alter reporter-*grumpy* activity (Fig. 3 K). We similarly analyzed the Tb927.10.11580-Tb927.10.11590 intergenic region (containing *KS17gene_3091a*), as Tb927.10.11580 tagging proved unfeasible. This region also inhibited reporter activity (~ 2-fold in bloodstream and ~ 5-fold in procyclic forms; Fig. 3 J), but unlike *grumpy*, did show significant DRBD3-dependent regulation, particularly in bloodstream forms, where luciferase activity decreased ~ 2-fold upon DRBD3 depletion (Fig. 3 K).

## Regulation of protein levels by alternative trans-splicing

**Fig. 4:**
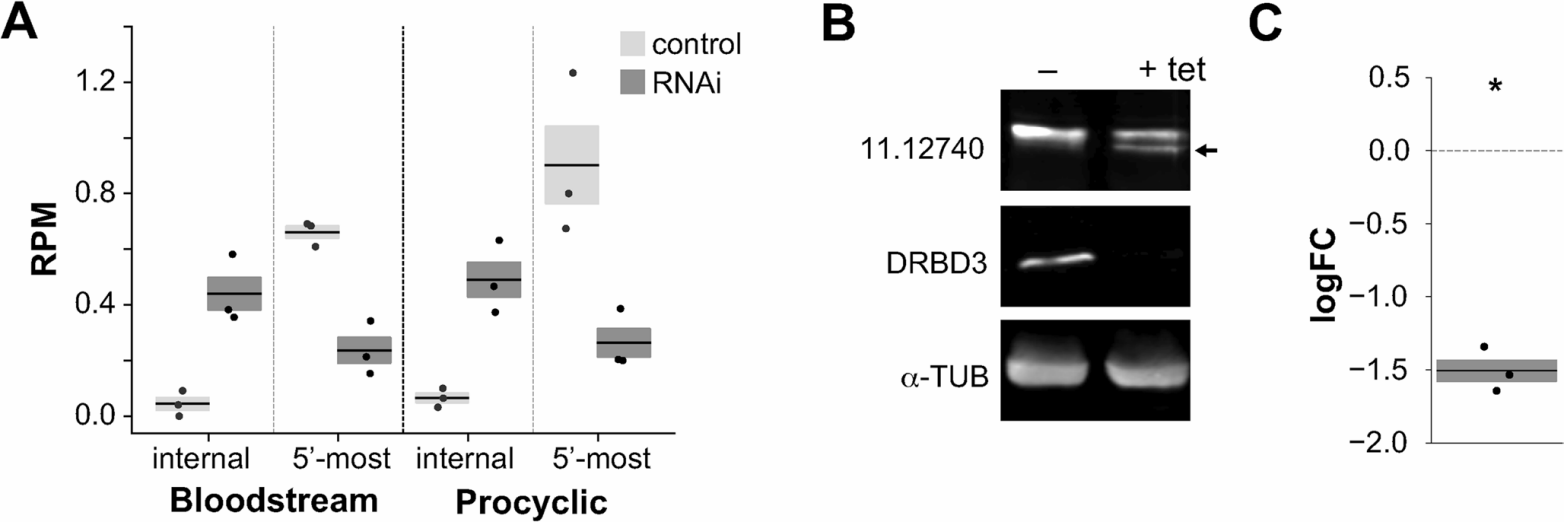
DRBD3 regulates the alternative *trans*-splicing of the transcript encoding the tRNA-import complex protein Tb927.11.12740. **A** Reads containing the spliced-leader at the internal SAS (splicing-acceptor site within the ORF) or the 5’ SAS (defining the longest transcript isoform). Values (reads per million mapped reads, RPM) are expressed as the mean ± s.e.m. (*n* = 3); all edgeR FDR values < 5 × 10^−4^. **B** Western blot analysis of Tb927.11.12740-TAP protein levels in uninduced vs DRBD3-silenced trypanosomes; α-tubulin was used as a loading control. The arrow indicates a truncated Tb927.11.12740-TAP species; the full-length protein has an estimated molecular mass of ~ 60 kDa. **C** Quantification of Tb927.11.12740-TAP depletion (full-length species). Log_2_FC (western signal in RNAi vs uninduced) values are shown as mean ± s.e.m. (*n* = 3). *, *p* < 5 × 10^−3^ (two-tailed, one-sample t-test vs log_2_ = 0, dashed line).

Some protein-coding genes in *T. brucei* are predicted to undergo alternative *trans*-splicing (ATS) within their coding sequences (https://tritrypdb.org/ [55]). ATS has the potential to generate N-truncated protein isoforms with distinct functional properties, including altered subcellular localization, stability or interaction networks [55], but no *trans*-acting regulators of these splicing events have been identified. Given that, as shown above, DRBD3 seems to control ATS of lncRNAs, we investigated whether it similarly regulates internal splice-acceptor site (SAS) usage within open-reading frames. To that end, we identified SAS located between the first and the second in-frame ATG of transcripts annotated as potentially encoding alternative open-reading frames (https://tritrypdb.org/), and compared SAS usage (number of reads containing the spliced-leader) in control and DRBD3-depleted transcriptomes. As shown in Table S11, we identified three transcripts that showed significant changes (|logFC|≥1.0 and FDR ≤ 0.01) in the number of reads containing the spliced-leader at internal SAS, and that were not present in equivalent analyses of DRBD18- or ZC3H41-depleted transcriptomes: a lysyl-tRNA synthetase (Tb927.8.1600), a mitochondrial leucine-rich repeat protein (LRRP, Tb927.6.1490) and a component of the mitochondrial tRNA import complex (Tb927.11.12740). Interestingly, these transcripts showed opposite regulation at the 5’-most SAS (which defines the longest transcript isoform) compared to the internal sites (Table S11). For instance, in the transcript encoding the mitochondrial tRNA complex component, spliced-leader reads in DRBD3-depleted cells were ~ 8-fold more abundant at internal SAS, whereas they decreased ~ 4-fold at the 5’-most SAS (Fig. 4 A and Table S11); overall transcript abundance was unaltered (Table S11 and Fig. S5A). These differential *trans*-splicing events would lead to the production of a truncated protein lacking the mitochondrial targeting signal (Figure S5B). To confirm regulation at the protein level, we TAP-tagged the protein at the C-terminus in procyclic cells, and its levels were monitored by western blot in uninduced *versus* DRBD3-silenced cells. As shown in Fig. 4B C, DRBD3 silencing promoted the formation of a truncated protein species while reducing full-length protein levels by approximately threefold.

## New insights into the regulatory role of DRBD3 in cis-splicing

**Fig. 5:**
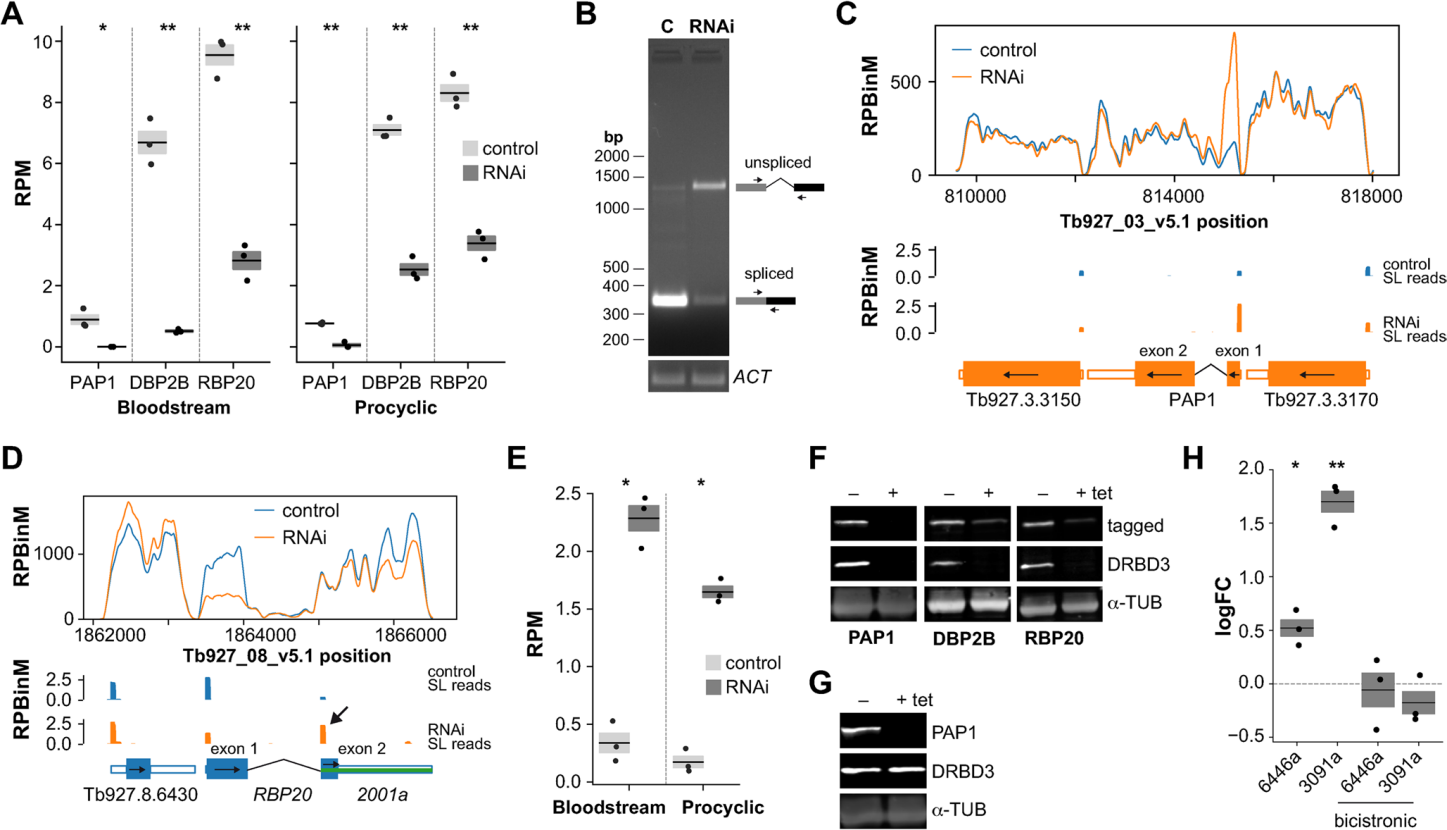
DRBD3 regulates the expression of intron-containing genes. **A** Quantification of split-reads corresponding to *PAP1*, *DBP2B* and *RBP20* transcripts in control vs DRBD3-depleted trypanosomes. Reads per million mapped reads (RPM) values are shown as mean ± s.e.m. of RNA-seq replicates (n = 3); *, p < 5 × 10^−2^, **, p < 5 × 10^−3^ (two-tailed, independent t-tests). **B** RT-PCR splicing assay of the *RBP20* transcript. Diagrams corresponding to unspliced (top) or spliced (bottom) transcripts are shown on the right; arrows represent the oligonucleotides used for PCR amplification. *Actin* (*ACT*) transcript was amplified using specific oligonucleotides to ensure equivalent amounts of input RNA in both samples. **C** and **D** coverage plots corresponding to the *PAP1* (procyclic) or *RBP20* (bloodstream) *loci*; the arrow in D points to spliced-leader reads at the 5’-end of *RBP20* exon 2. See Fig. 2 legend for details and Fig. S7 for additional plots. **E** Quantification of reads containing the spliced-leader at the 5’-end of *RBP20* exon2. RPM values are shown as mean ± s.e.m. of RNA-seq replicates (*n* = 3); both edgeR FDR values were < 5 × 10^−15^. **F** Expression of TAP-PAP1, 4xTy-DBP2B and 4xTy-RBP20 tagged proteins in DRBD3-depleted cells; α-tubulin was used as loading control; **G** Effect of PAP1 depletion on DRBD3 expression. **H** Effect of PAP1 depletion on the abundance of *KS17gene_6446a*, *KS17gene_3091a* and their respective bicistronic transcripts (see Fig. 2 for details). Fold changes (FC, log_2_ converted) relative to control of three independent RNAi inductions in procyclic cells are expressed as the mean ± s.e.m; *, *p* < 5 × 10^−2^, **, *p* < 5 × 10^−3^ (two-tailed, one-sample t-tests vs log_2_ = 0, dashed line)

DRBD3 has previously been proposed to regulate *cis*-splicing of the transcripts encoding the poly(A)-polymerase PAP1 and the DEAD-box RNA helicase *DBP2B* [11]. To confirm this, and to assess whether DRBD3 also regulates the recently identified *cis*-splicing event in the transcript coding for the RNA-binding protein 20 (RBP20 [6]), we quantified split reads (i.e., reads spanning two exons) in control and DRBD3-depleted transcriptomes (Fig. 5A). The number of split reads in control samples varied among the three transcripts, reflecting the relative abundance of the corresponding proteins (Fig. S6A). Notably, DRBD3 depletion led to a significant reduction in split reads for all three transcripts, indicating that *cis*-splicing is impaired in the absence of DRBD3. Inhibition of *RBP20 cis*-splicing was further validated by RT-PCR: as shown in Fig. 5B, DRBD3 silencing resulted in increased levels of the unspliced precursor and a concomitant decrease in mature transcript abundance. These findings provide direct experimental evidence for *cis*-splicing of the *RBP20* transcript in trypanosomes.

Examination of coverage plots corresponding to the three intron-containing genes revealed additional and noteworthy effects of DRBD3 depletion (Fig. 5C and Fig. S6B-D). Most strikingly, there was a marked increase in the number of reads corresponding to the first exon of *PAP1*, particularly in procyclic forms (Fig. 5C and Fig. S6B). This pattern closely resembled the upregulated lncRNAs profiles described above, and suggests that *PAP1* transcripts containing only the first exon are independently processed and polyadenylated in the absence of DRBD3. This was confirmed using 3’-nested RT-PCR (Fig. S6E-F), which amplified transcript isoforms comprising the first exon polyadenylated within the intronic sequence specifically upon DRBD3 silencing. Translation of transcripts containing only the first exon would produce a ~ 30 kDa tagged peptide, but this product was not detected by western blot (Fig. S6G), suggesting that such transcripts are not efficiently translated or the peptide is rapidly degraded.

*DBP2B* and *RBP20* coverage plots indicated the presence of transcripts isoforms containing the spliced leader (SL) sequence attached to the 5′ end of the second exon (Fig. 5D and Fig. S6C-D). In fact, a transcript comprising the second exon of *RBP20* was previously annotated as a lncRNA (*KS17gene_2001a*, Fig. 5D) [20]. To investigate this further, we quantified SL-exon2 reads in the three intron-containing RNAs; however, sufficient reads were only detected for RBP20. As shown in Fig. 5E, DRBD3 depletion led to a 6-to 8-fold increase in the number of RBP20 SL-exon2 reads in both life cycle forms.

Our differential expression analysis revealed distinct effects of DRBD3 depletion on the levels of *PAP1*, *DBP2B* and *RBP20* transcripts. In RNAi samples, exon 1 reads were significantly more abundant in *PAP1* and less abundant in *DBP2B* and *RBP20*, whereas reads corresponding to exon 2 were significantly altered only in *DBP2B* (Tables S2 and S3, Figs. 5C-D, S6B-D). To assess the consequences at the protein level, we generated cell lines that expressed N-terminal tagged versions of each protein and analyzed their levels upon DRBD3 depletion. As shown in Fig. 5F, all three proteins were markedly reduced in DRBD3-silenced cells. These results suggest that in the absence of DRBD3, *cis*-splicing is inhibited by competing *trans*-splicing events at cryptic sites. They strongly support the existence of cross-talk between *cis*- and *trans*-splicing, and highlight a pivotal role for DRBD3 in coordinating these RNA-processing pathways.

It was previously reported that PAP1 polyadenylated ncRNAs in procyclic *T. brucei*, and that its depletion led to increased levels of ~ 60 lncRNAs [39]. Since most lncRNAs had not yet been annotated at the time of that study, we reanalyzed the transcriptome of PAP1-depleted cells using updated lncRNAs annotations. We confirmed that the vast majority (83%) of all upregulated transcripts (coding plus lncRNAs) in PAP1-silenced cells corresponds to lncRNAs (Fig. S6H). We next compared the differentially expressed lncRNA profiles in PAP1- and DRBD3-depleted cells, and found that 70 of the 99 DRBD3-upregulated lncRNAs were also upregulated in the PAP1 dataset, including those examined in this study (Table S12 and Fig. S6I). These findings suggest that the increased abundance of lncRNAs observed upon DRBD3 silencing is largely a consequence of DRBD3-dependent PAP1 downregulation. To test the inverse scenario (whether PAP1 depletion alters DRBD3 levels), we generated an RNAi cell line expressing dsRNA against the PAP1 transcript. PAP1-silenced cells showed only minor growth defects (Fig. S6J). As shown in Fig. 5G, PAP1 protein was efficiently depleted upon RNAi induction, whereas DRBD3 levels remained unchanged. We also tested for potential interaction between DRBD3 and PAP1 using pull-down assays, but no association was detected (Fig. S6K). Finally, we examined whether alternative *trans*-splicing of lncRNAs *KS17gene_6446a* and *KS17gene_3091a* was affected in PAP1-depleted cells. Although coverage profiles revealed increased abundance of both lncRNAs, the patterns differed from those observed in DRBD3-silenced cells (Fig. S6L-M). Indeed, quantitative RT-PCR assays showed no changes in the in the levels of the corresponding bicistronic species (Fig. 5H). Thus, our results indicate that DRBD3 acts upstream PAP1 in controlling lncRNAs expression.

## Discussion

The results presented in this study reveal novel mechanisms by which DRBD3 regulates gene expression in *Trypanosoma brucei*. We first demonstrated that silencing DRBD3 expression leads to a significant increase in the abundance of a specific cohort of lncRNAs. Our findings are consistent with a role for DRBD3 in repressing the use of cryptic splice sites which, when utilized, result in the accumulation of lncRNAs and mRNAs with truncated 3′ untranslated regions (3′-UTRs), a mechanism reminiscent of that proposed for DRBD18 [37, 50]. This interpretation is well supported by tethering assays showing that DRBD3 can inhibit *trans*-splicing when positioned at a splicing polypyrimidine tract upstream of a reporter gene [16]. It further reinforces the view that DRBD3 is the homologue of mammalian PTB1 and supports the hypothesis that the role of PTB1 in splice site selection may have already been present in the last eukaryotic common ancestor [11, 16].

Transcripts with shortened 3′-UTRs may be misregulated due to the loss of *cis*-regulatory elements. Although we detected altered expression of some neighboring genes in DRBD3-depleted cells, our data suggest that this is not a general effect, at least at the RNA level. This raises questions about the functional significance of the DRBD3-regulated lncRNAs. One possibility is that they are non-functional by-products of aberrant *trans*-splicing and thus have no major role in parasite physiology. This view is supported by the observation that PAP1, whose depletion also results in lncRNA accumulation, is not essential for cell viability ([56, 57] and this study), although another study reported impaired growth upon PAP1 silencing [39]. However, the discovery that *grumpy* and *TblncRNA-23* play regulatory roles in gene expression and parasite differentiation highlights the possibility that other DRBD3-regulated lncRNAs may likewise have important, yet unidentified, functions. The roles of *grumpy* and *TblncRNA-23* were inferred from overexpression experiments [20, 25]. Upregulation of these lncRNAs in DRBD3-silenced cells did not reach the levels observed in those studies, which may explain the modest effects we observed, for instance, on procyclins regulation. In general, DRBD3-dependent regulation of lncRNA abundance seems similar in bloodstream and procyclic trypanosomes, although some differences are evident. These stage-specific patterns suggest additional layers of regulation, but they were not explored further in this study and await experimental validation.

The role of DRBD3 in masking cryptic splice sites extends to protein-coding genes as well. Our data indicate that DRBD3 prevents *trans-*splicing at an internal cryptic site within Tb927.11.12740, a gene encoding a component of the mitochondrial tRNA import complex. When this site is used, a truncated, likely non-functional isoform is produced, accompanied by a marked reduction in the full-length protein. While alternative *trans*-splicing is not considered a major driver of proteomic diversity in trypanosomes [58], the regulation of such events by DRBD3 may nonetheless be essential for maintaining cellular homeostasis. Supporting this notion, Tb927.11.12740 depletion has been shown to impair cell growth [59].

DRBD3 has previously been implicated in the regulation of *cis*-splicing for *PAP1* and *DBP2B* transcripts [11], and in this study, we provided experimental evidence supporting *cis*-splicing of the recently identified intron in *RBP20*, which also appears to be regulated by DRBD3. Consistent with this, DRBD3 depletion led to a marked reduction in the protein levels of all three genes, most notably PAP1. Importantly, the majority of lncRNAs affected by DRBD3 silencing were also differentially expressed in PAP1-depleted cells, raising the possibility that their upregulation might be an indirect consequence of PAP1 loss. However, the DRBD3-dependent alternative *trans-*splicing events characterized in this study were not detected upon PAP1 depletion, supporting a more direct role for DRBD3 in regulating lncRNA abundance. A model has been proposed in which an RNA-binding protein coordinates the recruitment of both PAP1 and the RNA processing machinery to specific lncRNA substrates [39]. Although DRBD3 could fulfill such a role, we found no evidence of a physical interaction between DRBD3 and PAP1. Still, we cannot exclude transient or RNA-mediated interactions that may be disrupted during immunoprecipitation, as observed for some metazoan splicing complexes [60].

Remarkably, we found that the absence of DRBD3 leads to aberrant processing events, including polyadenylation within the *PAP1* intron and increased *trans*-splicing of exon 2 from the *RBP20* transcript. These observations strengthen the idea of interplay, or even competition, between *cis*- and *trans*-splicing in trypanosomes, as previously proposed [4, 6], and highlight DRBD3 as a key factor in mediating this regulatory balance. Interestingly, mammalian PTB1 inhibits intron inclusion by binding to the exon, or at the 3’ splice site [61], and DRBD3 has been shown to associate with the *PAP1* intron [11, 14]. This suggests a mechanism in which DRBD3 promotes *cis*-splicing by preventing the *trans*-splicing of individual exons in intron-containing transcripts.

DRBD3 has been previously characterized as a key regulator of mRNA abundance, transport, and translation, and as a mechanistic link between metabolic regulation and the response to starvation stress [10, 11, 14, 15, 62]. In this study, we have uncovered additional roles for DRBD3 in modulating lncRNA abundance, alternative open reading frame usage, and the balance between *cis*- and *trans*-splicing. *Cis*-splicing in trypanosomes has been proposed to function as a mechanism for ‘regulating the regulators’ [6], and we have shown in this study that DRBD3 is crucial to maintain proper levels of the proteins encoded by *cis*-spliced transcripts. Our results position DRBD3 as a key upstream regulator in the gene expression hierarchy of trypanosomes, acting as a master regulator of both coding and non-coding gene expression.

## Supplementary Information

Below is the link to the electronic supplementary material.

Supplementary Material 1

Supplementary Material 2

Supplementary Material 3

Supplementary Material 4

Supplementary Material 5

Supplementary Material 6

Supplementary Material 7

## Data Availability

The RNA-seq data discussed in this publication are accessible are available at the NCBI’s Gene Expression Omnibus repository, https://www.ncbi.nlm.nih.gov/geo/query/acc.cgi?acc=GSE272310.
