# Supplementary material for: DRBD3 regulates long non-coding RNA abundance and cryptic splice site selection in trypanosomes
Source: Cell Mol Life Sci. 2025 Nov 6;82(1):386. doi: 10.1007/s00018-025-05929-w (PMC12592628; doi:10.1007/s00018-025-05929-w)
Supplement: Supplementary file 2 — Supplementary Material 2 [file 18_2025_5929_MOESM2_ESM.pdf]

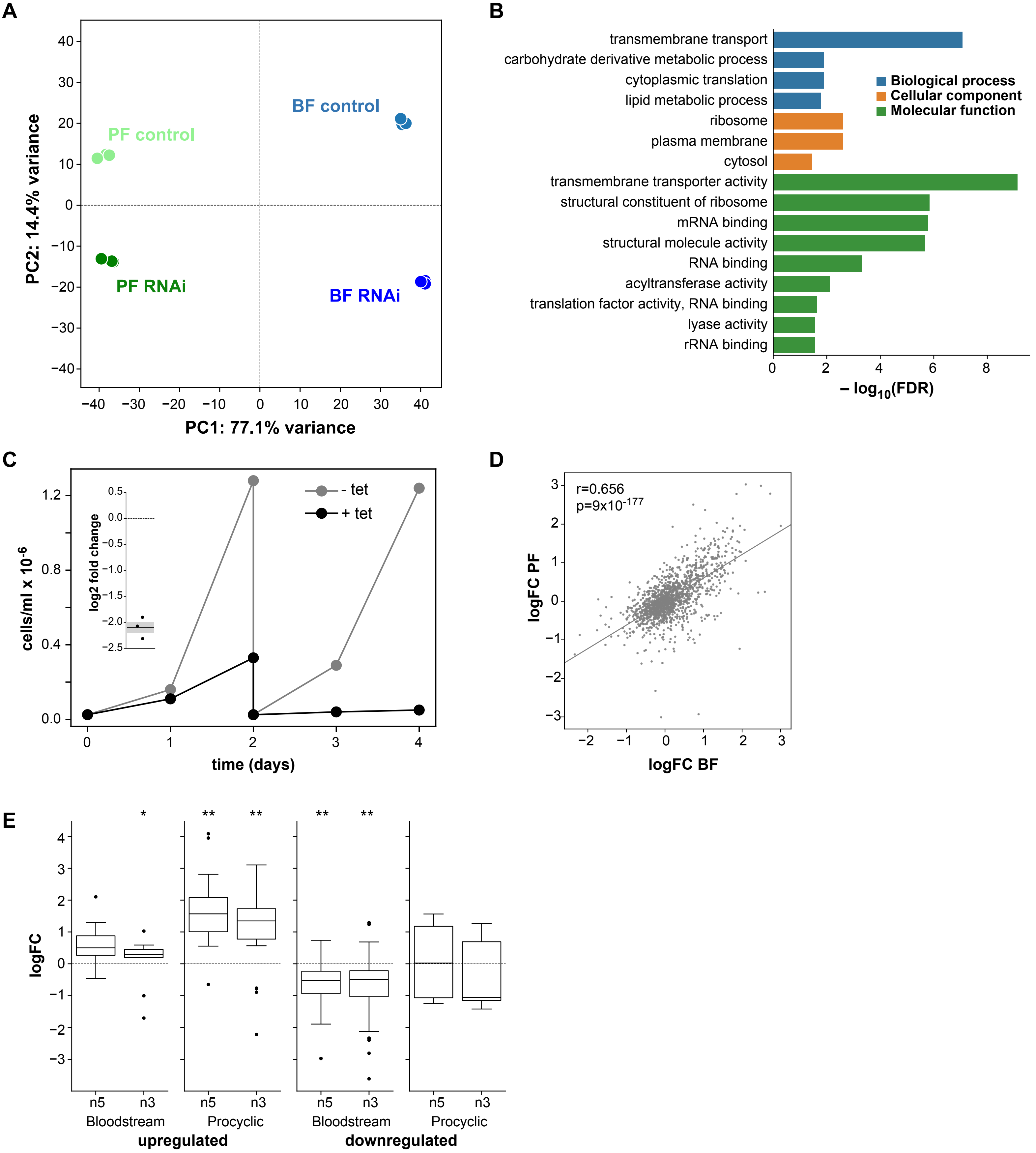

Supplementary Fig S1 A Principal component analysis of RNA-seq datasets. Each point represents a replicate. BF, bloodstream form; PF, procyclic form; PC, principal component. **B** Gene Onthology analysis of proteins encoded by transcripts downregulated in DRBD3-depleted bloodstream forms. **C** Effect of ZC3H41 depletion on bloodstream cells growth. Single-marker S16 trypanosomes were transfected with plasmid pGR309, which expresses double-stranded RNA (dsRNA) corresponding to *ZC3H41* in a tetracycline (tet)-inducible fashion (Ceballos-Pérez, 2023). Cell cultures were followed for up 4 days and diluted at day 2. Depletion of *ZC3H41* transcript was confirmed by quantitative RT-PCR after 2 days of tet induction (inset); fold changes (log2 converted, logFC) are expressed as the mean (horizontal line) ± SEM (shaded area) of three independent RNAi inductions in bloodstream trypanosomes. The horizontal dashed line indicates log2 = 0, i.e. no change in gene expression. **D** Correlation analysis of fold-change values (log2 converted, logFC) of all annotated lncRNAs in BF vs PF RNA-seq datasets. Pearson correlation coefficient and respective p-value are shown. **E** Expression changes in protein-coding genes adjacent to DRBD18-regulated lncRNAs. Boxplots represent log2 fold-change values (logFC) of 5´-neighbors (n5) and 3´-neighbors (n3). Two-tailed, one-sample t-tests assessed whether mean values differed significantly from log2 = 0 (no expression change, dashed line). *, $p<5x10^{-2}$; **, $p<5x10^{-3}$
