# Supplementary material for: DRBD3 regulates long non-coding RNA abundance and cryptic splice site selection in trypanosomes
Source: Cell Mol Life Sci. 2025 Nov 6;82(1):386. doi: 10.1007/s00018-025-05929-w (PMC12592628; doi:10.1007/s00018-025-05929-w)
Supplement: Supplementary file 3 — Supplementary Material 3 [file 18_2025_5929_MOESM3_ESM.pdf]

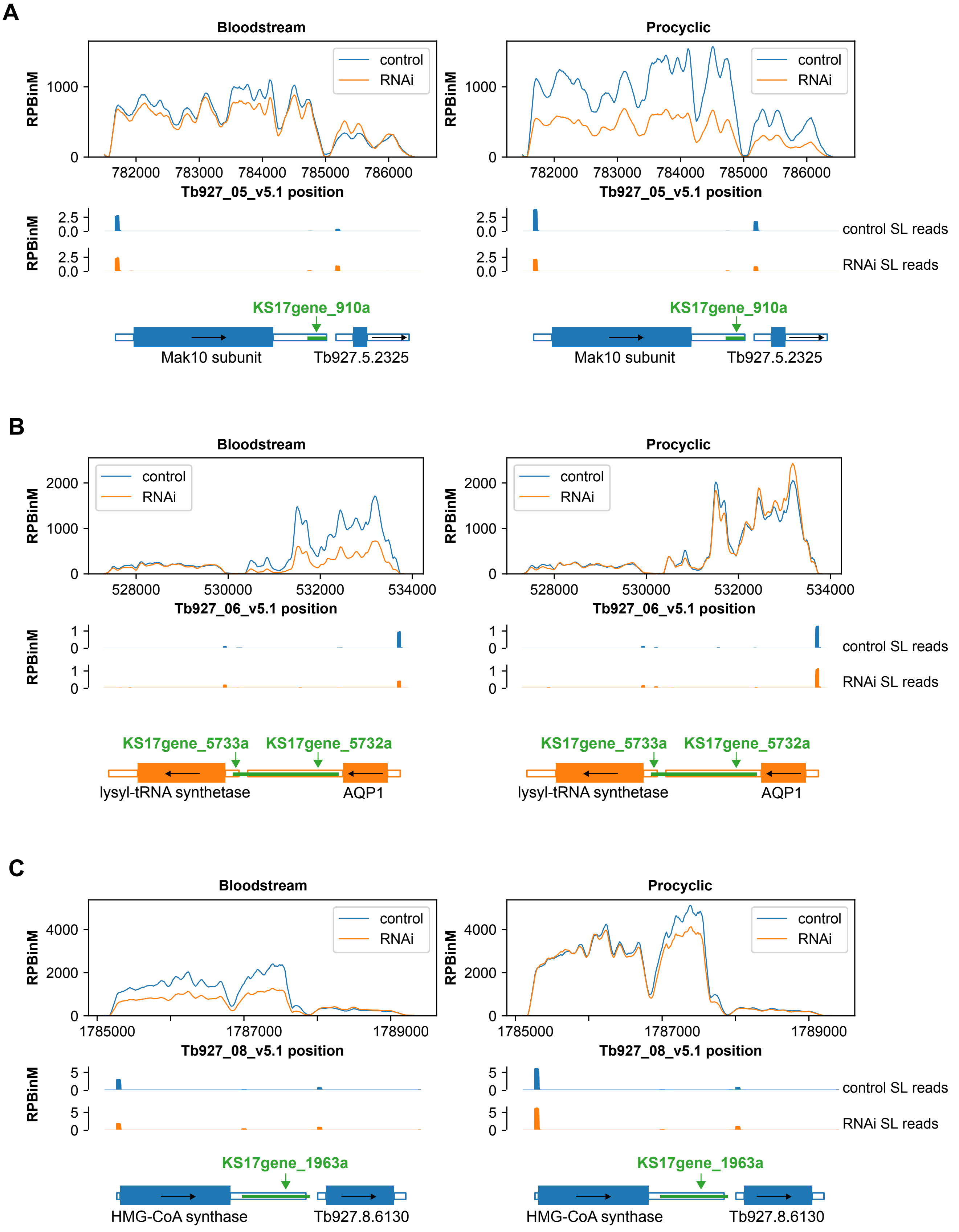

Supplementary Fig. S2 Coverage plots of representative downregulated lncRNAs and flanking protein-coding genes in control (blue profiles) vs DRBD3-depleted (orange profiles) bloodstream and procyclic trypanosomes. Average read counts across replicates were obtained using sliding windows (bin size, 100 bp; step, 10 bp) and normalized to library size (RPBinM, reads per bin per million mapped reads). Profiles corresponding to reads containing the spliced-leader sequence (SL) are also shown. Open-reading frames are represented as thick, filled boxes (blue, genes in the Watson (+) strand; orange, genes in the Crick (–) strand), whereas untranslated regions are depicted as thin, empty boxes. Long-non coding RNAs are shown as thin green filled boxes. Black arrows indicate the direction of transcription. **A** KS17gene_910a *locus*. **B** KS17gene_5732a/KS17gene_5733a *locus*.
