# Supplementary material for: DRBD3 regulates long non-coding RNA abundance and cryptic splice site selection in trypanosomes
Source: Cell Mol Life Sci. 2025 Nov 6;82(1):386. doi: 10.1007/s00018-025-05929-w (PMC12592628; doi:10.1007/s00018-025-05929-w)
Supplement: Supplementary file 4 — Supplementary Material 4 [file 18_2025_5929_MOESM4_ESM.pdf]

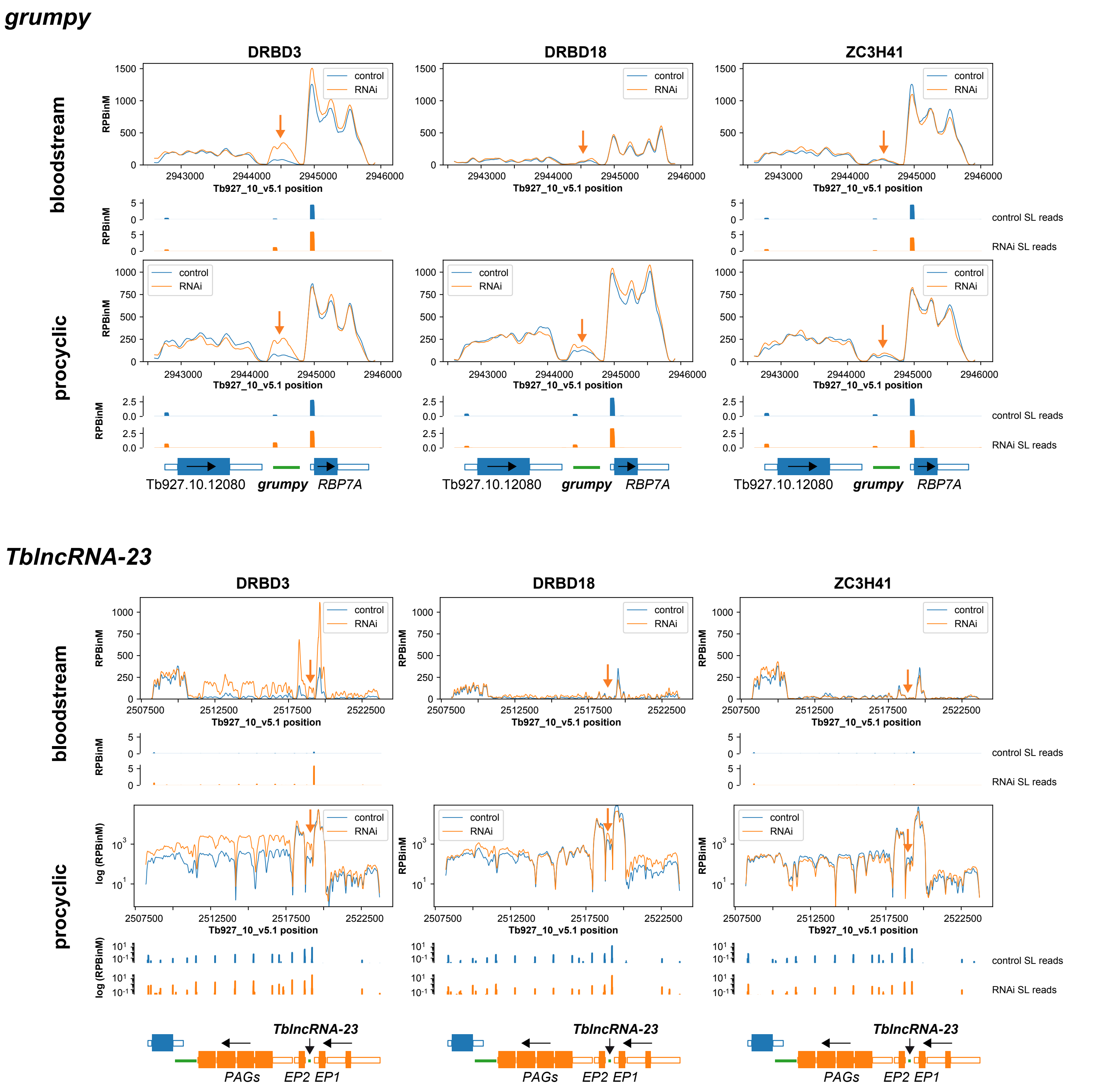

Supplementary Fig. S3 Regulation of *grumpy* and *TblncRNA-23* expression in DRBD3-, DRBD18- and ZC3H41-depleted bloodstream and procyclic trypanosomes. Average read counts across replicates were obtained using sliding windows (bin size, 100 bp; step, 10 bp) and normalized to library size (RPBinM, reads per bin per million mapped reads). Profiles corresponding to reads containing the spliced-leader sequence (SL) are also shown. Protein-coding genes are represented as thick boxes (blue, genes in the Watson (+) strand; orange, genes in the Crick (–) strand), whereas long-non coding RNAs are shown as thin green boxes. Black arrows indicate the direction of transcription. RPbinM values for *TblncRNA-23* coverage plots are displayed on a log10 scale. Relevant lncRNA names are highlighted in bold, and their positions indicated by orange vertical arrows above the corresponding coverage profiles
