# Supplementary material for: DRBD3 regulates long non-coding RNA abundance and cryptic splice site selection in trypanosomes
Source: Cell Mol Life Sci. 2025 Nov 6;82(1):386. doi: 10.1007/s00018-025-05929-w (PMC12592628; doi:10.1007/s00018-025-05929-w)
Supplement: Supplementary file 5 — Supplementary Material 5 [file 18_2025_5929_MOESM5_ESM.pdf]

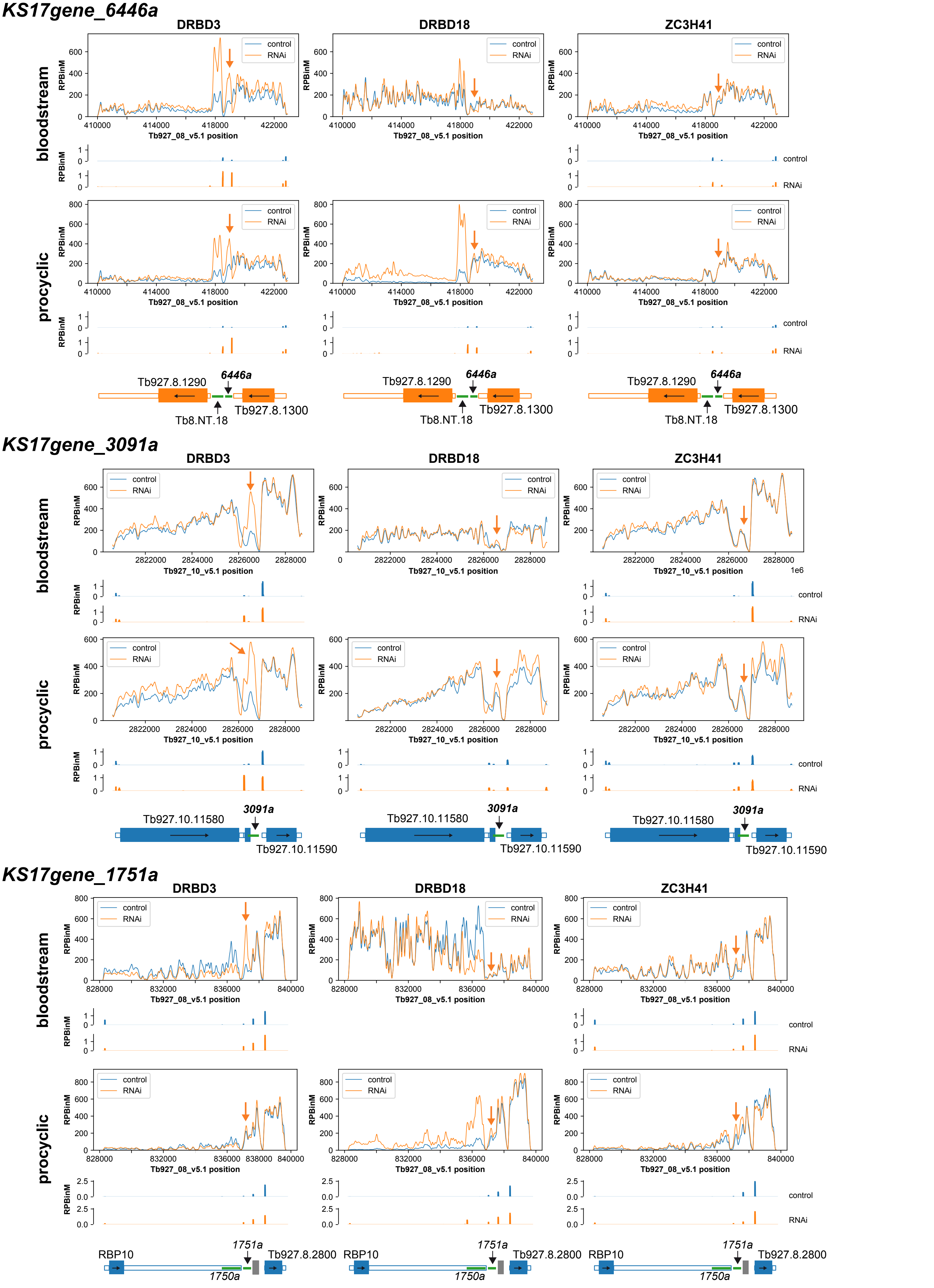

Supplementary Fig S4 Regulation of *KS17gene_6446a*, *KS17gene_3091a* and *KS17gene_1751a* expression in DRBD3-, DRBD18- and ZC3H41-depleted bloodstream and procyclic trypanosomes. See Fig S3 legend for details.
