# Supplementary material for: DRBD3 regulates long non-coding RNA abundance and cryptic splice site selection in trypanosomes
Source: Cell Mol Life Sci. 2025 Nov 6;82(1):386. doi: 10.1007/s00018-025-05929-w (PMC12592628; doi:10.1007/s00018-025-05929-w)
Supplement: Supplementary file 6 — Supplementary Material 6 [file 18_2025_5929_MOESM6_ESM.pdf]

A

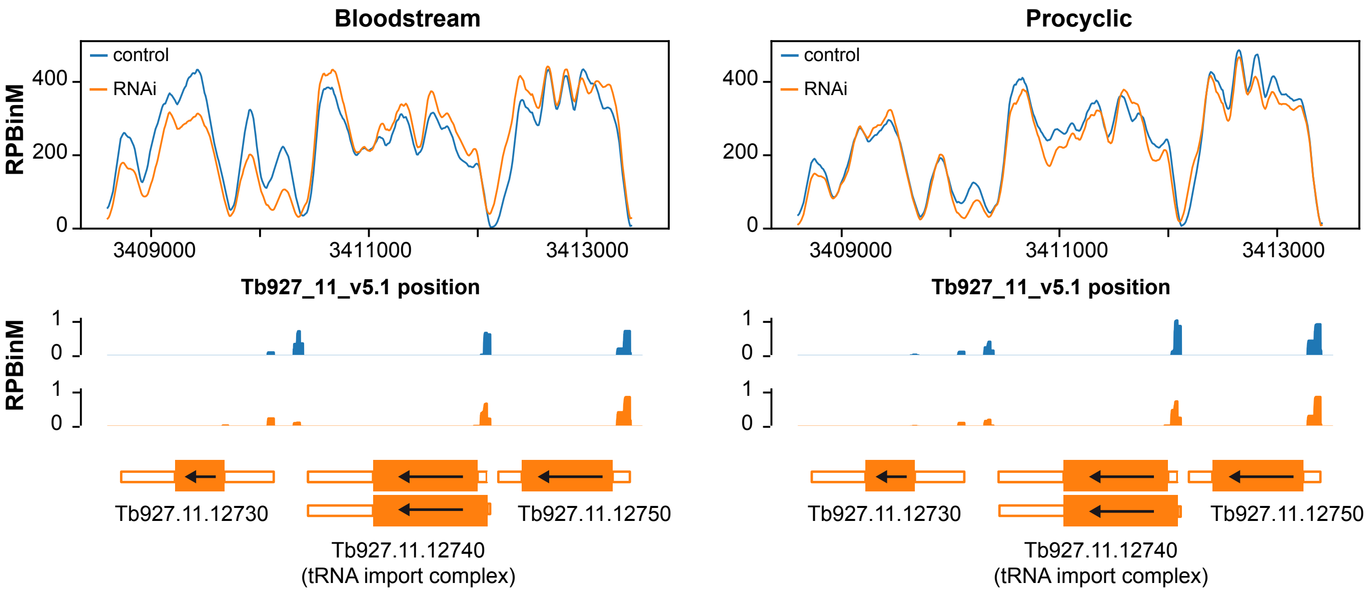

B

tRNA import **M**RRTCCVYRRVLRRHWANTTVTEGPSQLSS**M**QSAVSLSGGMCGEASSLAM

LRRP **M**CSNPGGISCALFTCITSYIAQQPHENTPWVWYATHPRSALA**M**IATPSKQ

LysRS **M**WICVFFFFLRPEVEVRTLQFYSLAPTSLFFLPSPLLLIVVVFFTIGNVAIHKPRFYLCTVGKGKKKQENIRAQET**M**SAVEELRA

Supplementary Fig S5 DRBD3-dependent regulation of alternative *trans*-splicing within ORFs.
A Coverage plot corresponding to the mitochondrial tRNA import component Tb927.11.12740. See Fig. 2 legend for details.
B N-terminal amino acid sequences of proteins whose transcripts undergo alternative *trans*-splicing. Sequences between alternative initiator metionines (highlighted in bold) are shown in grey. Arrows indicate the position of the internal splicing-acceptor site within the transcript. GeneIDs are: tRNA import complex, Tb927.11.12740; leucine-rich repeat protein (LRRP), Tb927.6.1490; lysyl tRNA synthethase (LysRS), Tb927.8.1600.
