# Supplementary material for: DRBD3 regulates long non-coding RNA abundance and cryptic splice site selection in trypanosomes
Source: Cell Mol Life Sci. 2025 Nov 6;82(1):386. doi: 10.1007/s00018-025-05929-w (PMC12592628; doi:10.1007/s00018-025-05929-w)
Supplement: Supplementary file 7 — Supplementary Material 7 [file 18_2025_5929_MOESM7_ESM.pdf]

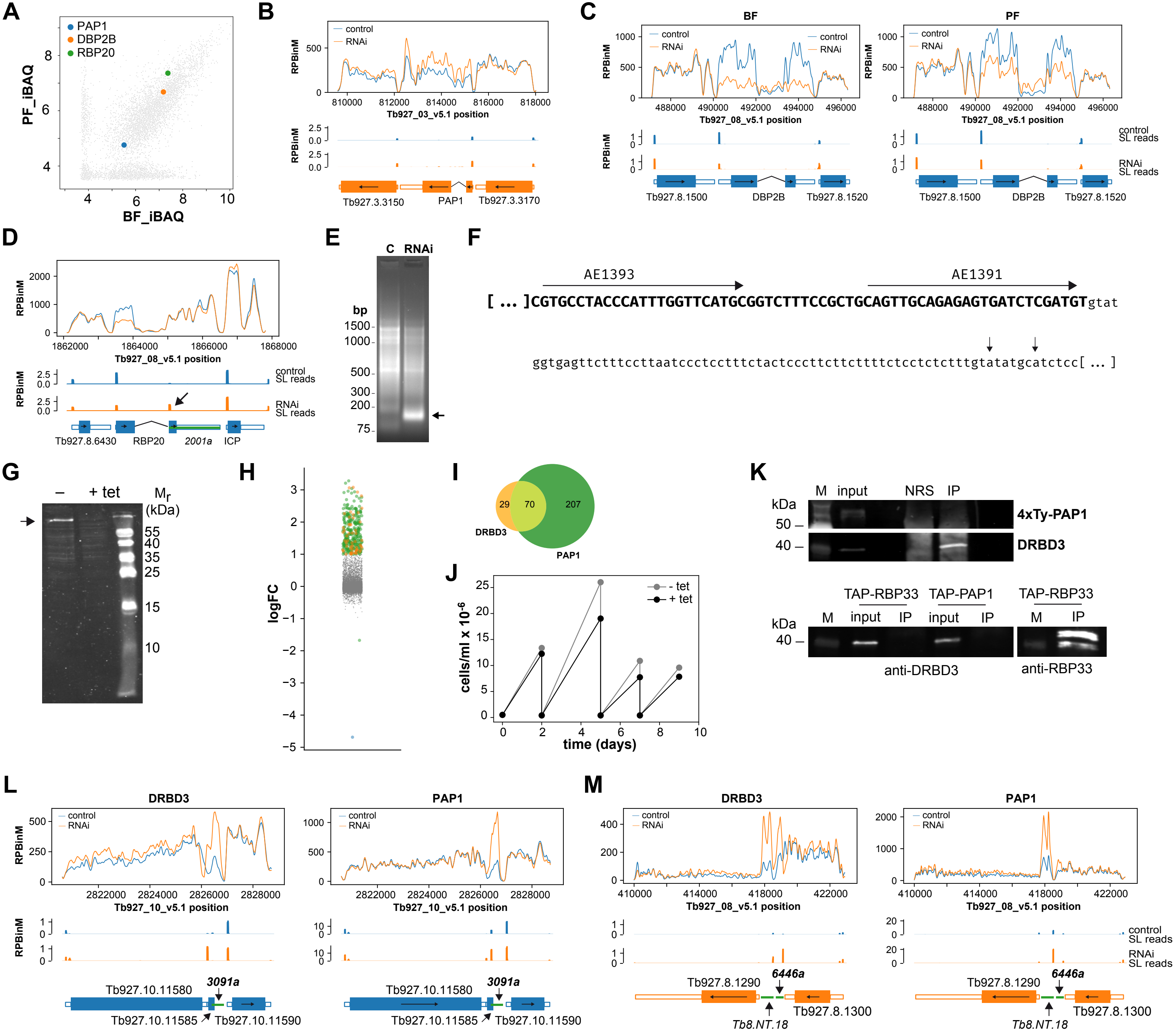

Supplementary Fig. S6 Supplementary material corresponding to Fig. 5. **A** Protein abundances of PAP1, DBP2B and RBP20. Data from deep proteome surveys carried out in bloodstream (BF) and procyclic (PF) strain 927 trypanosomes were plotted according to protein iBAQ (intensity-based absolute quantification) values (Tinti & Ferguson, 2022). Each dot represents a protein. **B**, **C** and **D** coverage plots corresponding to *PAP1* (bloodstream), *DBP2B* (bloodstream and procyclic) and *RBP20* (procyclic forms) *loci*. See Fig. 2 legend for details. The arrow in D points to spliced-leader reads at the 5'-end of RBP20 exon 2. **E** 3'-nested RT-PCR using oligo(dT) and PAP1 exon 1 primers. The arrow indicates the amplicon corresponding to the polyadenylated exon 1. **F** Polyadenylation of a *PAP1* transcript species containing only the first exon. The 3'-end sequence of exon 1 is represented in bold uppercase along with the oligonucleotides used for 3'-nested RT-PCR. The 5'-end sequence of the intron is indicated in lowercase; vertical arows indicate polyadenylation sites. **G** A TAP-tagged PAP1 peptide corresponding to translation of the first exon was not detected by western blot (expected size ~30 kDa). Protein extracts from uninduced or DRBD3-silenced cells were separated by 15% SDS-PAGE. The arrow indicates full-length PAP1. **H** Strip plot representing log2 ratios of PAP1 RNAi RPMK values relative to control. Grey dots indicate transcripts with |logFC| values < 1.0. Blue dots represent downregulated mRNAs (logFC<-1.0); orange dots, upregulated mRNAs (logFC>1.0); green dots, lncRNAs (|logFC|>1.0). **I** Venn diagram of upregulated lncRNAs (logFC>1.0) detected upon depletion of DRBD3 (orange) and PAP1 (green). Numbers indicate unique and common lncRNAs between the datasets. **J**, Effect of PAP1 depletion on procyclic trypanosomes growth. A cell line expressing a TAP-tagged version of PAP1 was transfected with plasmid pGR317, which produces double-stranded RNA corresponding to the second exon of *PAP1* in a tetracycline (tet)-inducible fashion. Cell cultures were followed for up 9 days and diluted as necessary. PAP1 depletion was confirmed by western blot (see Fig. 5F). **K**, Absence of detectable interaction between DRBD3 and PAP1. Top panel, protein extracts from cells expressing 4xTy-PAP1 were immunoprecipitated with anti-DRBD3 antibodies or normal rabbit serum (NRS); DRBD3, but not PAP1, was detected in the immunoprecipitated sampes (IP); M, size markers. Bottom panel, TAP-PAP1 was purified using IgG magnetic beads, and eluted using tobacco-etch virus (TEV) protease. A cell line expressing TAP-RBP33 (Gómez-Liñán et al, 2022) was used as a control. DRBD3 was not detected in either TAP-RBP33 (negative control) or TAP-PAP1 purifications. RBP33 was readily detected in TAP-RBP33 samples (positive control). **L** and **M** coverage plots corresponding to the KS17gene_6446a and KS17gene_3091a *loci* in procyclic DRBD3 and PAP1 RNAi datasets. 5′ directed SL libraries (Chikne et al.,2017) were used for SL read coverages in PAP1 graphs. See Fig. 2 legend for details
